# A Metric-Driven Evaluation Framework for Remaining Useful Life Prognosis with Quantified Uncertainty

**DOI:** 10.3390/s26072230

**Published:** 2026-04-03

**Authors:** Govind Vashishtha, Sumika Chauhan, Merve Ertarğın

**Affiliations:** 1 Faculty of Geoengineering, Mining and Geology, Wroclaw University of Science and Technology, Na Grobli 15, 50-421 Wroclaw, Poland; govindyudivashishtha@gmail.com; 2 Department of Mechanical Engineering, Graphic Era Deemed to Be University, Dehradun 248002, India; 3 Department of Electrical and Electronics Engineering, University of Munzur, Tunceli 62000, Turkey; merveboydak@munzur.edu.tr

**Keywords:** remaining useful life, uncertainty quantification, long short-term memory, deep state space model, slime mold optimization

## Abstract

This paper introduces a novel metric-driven evaluation framework for Remaining Useful Life (RUL) prognosis in rotating machinery, featuring robust uncertainty quantification. Accurate RUL prediction is vital for optimizing maintenance and preventing failures, but existing methods often struggle with complex nonlinear degradation or lack reliable uncertainty estimates. Our proposed framework integrates a probabilistic Deep State Space Model (DSSM) with a variational inference approach to model complex, non-linear degradation trends and inherent aleatoric uncertainty. A key innovation is the use of the Slime Mold Algorithm (SMA) for efficient hyperparameter optimization, ensuring maximum accuracy. Furthermore, an online adaptation mechanism, governed by a heuristic reinforcement learning agent, allows the model to continuously update its knowledge and adapt to concept drift in real-time. Experimental validation on the IMS bearing dataset demonstrates superior RUL prediction accuracy, evidenced by the lowest Root Mean Square Error (RMSE) of 8.1829 cycles, and a PICP of 0.59416. This dual capability makes the framework highly suitable for real-world predictive maintenance, enhancing safety and reliability.

## 1. Introduction

Accurately forecasting the Remaining Useful Life (RUL) of rotating machinery, along with quantifying its associated uncertainties, is crucial for effective intelligent maintenance and ensuring operational reliability. Accurate RUL prediction can not only help optimize the maintenance strategy and reduce costs, but also effectively prevent sudden failures and enhance the safety of the equipment, thus improving the overall productivity and prolonging the service life of the equipment [1,2]. In the era of increasing industrial automation and intelligence, the accurate assessment and prediction of rotating machinery’s RUL, particularly amidst complex operational parameters and changing environmental factors, stands out as both a key research priority and a persistent challenge. Accurate RUL prediction is highly dependent on the degree of assessment of the degradation state of the mechanical system, and how to construct an accurate system degradation model and predict its RUL needs to be solved urgently [3,4,5].

Existing methods for RUL prediction of rotating machinery have certain shortcomings, especially in capturing the degradation trend over a long period of time and quantifying the uncertainty. Currently, RUL prediction methods for rotating machinery are mainly classified into three categories: physical model-based, and stochastic process-based approaches [6,7,8,9]. Physical model-based methods make predictions by constructing a physical degradation model of the system, but due to the complexity of physical modeling and the limitations of assumptions, such methods are difficult to adapt to complex nonlinear degradation processes, and thus gradually reveal their inadequacies [10].

With the rise of data-driven approaches, machine learning-based models (e.g., artificial neural networks (ANN), support vector machines (SVM), random forests (RF), etc., and convolutional neural networks (CNN) have been progressively applied to RUL prediction [11,12,13,14,15]. While CNNs excel at feature extraction, they often encounter difficulties when modeling long-term dependencies and complex, non-linear degradation behaviors. In contrast, Long Short-Term Memory (LSTM) networks have gained significant attention in RUL prediction research, precisely because of their superior ability to capture long-range temporal relationships and efficiently manage non-linear degradation processes. Researchers have explored various enhancements to LSTM for RUL prediction. For instance, Xiahou et al. [16] developed a Bayesian two-input LSTM (BDIC-LSTM) specifically to quantify RUL prediction uncertainty. Shi et al. [17] introduced a dual-attention LSTM (DA-LSTM) model, employing a soft-attention mechanism to better capture sequential degradation features. Similarly, Wang et al. [18] improved LSTM by integrating a multi-stage convolutional autoencoder (MSCAE), significantly enhancing RUL prediction accuracy. However, although data-driven methods improve the prediction accuracy, they usually lack the quantification of the uncertainty of the prediction results, which may pose some risks in practical applications [19].

To compensate for this deficiency, methods based on stochastic processes have been proposed, such as the Wiener process (WP), the gamma process and the generalized Cauchy process [20,21,22]. Among the methods for RUL uncertainty quantification, the Wiener process stands out as widely used, thanks to its continuous, Markovian, and Gaussian characteristics. While initial investigations centered on linear Wiener process models, which assumed a simplified, linear system degradation, these models were ultimately unable to capture the true nonlinear degradation patterns. This limitation prompted a critical shift, leading researchers to explore nonlinear Wiener process models for more accurate system lifetime forecasting. Wang et al. [23] modeled the multi-stage Wiener degradation process and improved the RUL prediction accuracy by Bayesian information criterion. He et al. [24] interval prediction based on improved conditional parameterized convolution and nonlinear Wiener process quantifies RUL uncertainty. Recent advances in trustworthy and physics-guided data-driven modeling represent an important emerging paradigm in RUL prognosis. Frameworks such as physics-guided degradation trajectory modeling incorporate domain knowledge as constraints within data-driven architectures, improving physical consistency of predictions. Similarly, trustworthy multistep-ahead RUL prediction frameworks address critical industrial requirements of reliability and explainability. While hybrid approaches combining deep learning with the Wiener process, such as those explored by Wang et al. [23] and He et al. [24], leverage stochastic process theory for uncertainty quantification, they rely on fixed parametric drift functions that limit flexibility for complex nonlinear degradation. In contrast, the proposed DSSM learns the degradation trajectory implicitly through variational inference, requiring no parametric assumptions about the degradation path. Yin et al. [25] integrated physics-guided fault-evolution knowledge with adversarial meta-learning to enable few-shot tool-state recognition; experiments that show superior accuracy in detecting healthy, worn, and broken tool conditions with minimal target data requirements.

While these methods have shown improved results, they still contend with challenges related to model complexity and the precision of uncertainty quantification. For instance: (1) Wiener process models, as currently implemented, often fail to capture complex nonlinear degradation trends because they depend on fixed drift functions (like power or exponential). (2) The high-dimensional parameter sets of contemporary hybrid models present a significant hurdle, as manual tuning becomes inefficient and yields suboptimal outcomes. (3) Crucially, rigorous uncertainty quantification for RUL predictions is largely absent in most data-driven methodologies.

In this paper, a novel metric-driven evaluation framework for RUL prognosis that explicitly incorporates quantified uncertainty is proposed. The key contributions include the development of a probabilistic DSSM coupled with a variational inference approach for robust degradation modeling, an efficient hyperparameter optimization strategy utilizing the Slime Mold Algorithm (SMA), and an adaptive online learning mechanism to counter concept drift. This comprehensive framework aims to deliver highly accurate RUL predictions alongside reliable uncertainty bounds, crucial for intelligent maintenance decisions in complex industrial settings.

The remainder of this paper is organized as follows: Section 2 provides the fundamental theoretical background of Long Short-Term Memory networks and Uncertainty Quantification. Section 3 details the proposed methodology, including the DSSM architecture, RUL estimation head, objective function, SMA optimization, and online adaptation strategy. Section 4 presents the experimental validation, covering dataset description, data preprocessing, feature extraction, and a thorough discussion of the results, including comparisons with other methods and an analysis of uncertainty quantification metrics. Finally, Section 5 concludes the paper.

## 2. Fundamental Theory

### 2.1. Long Short-Term Memory (LSTM)

**Figure 1:**
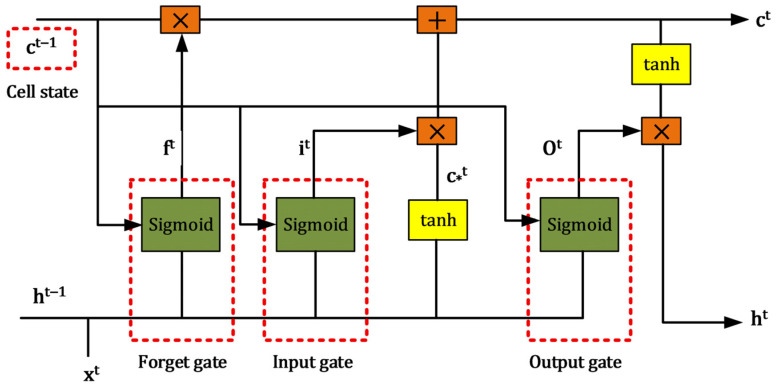
Basic architecture of LSTM.

An LSTM network comprises multiple LSTM units connected over time, forming a hierarchical structure designed to capture long-term dependencies in sequential data, which is crucial for modeling complex degradation trends [26]. As illustrated in Figure 1, the neural unit operates through a system of three specialized gates: the forget gate, the input gate, and the output gate.

First, the input gate and a candidate cell state are responsible for determining which new information from the current time step is added to the cell state. Specifically, the input gate ($i_{t}$) is calculated using a sigmoid activation function, often represented as Equation (1)

(1) $$i_{t}=\sigma \left(W_{i}\cdot \left[h_{t-1},x_{t}\right]+b_{i}\right)$$

where $W_{i}$ and $b_{i}$ are the weight and bias for the input gate, respectively, $h_{t-1}$ is the output from the previous time step, and $x_{t}$ is the current input. Simultaneously, a candidate cell state ($\tilde{C_{t}}$) is generated using a hyperbolic tangent (tanh) activation, given by Equation (2)

(2) $$\tilde{C_{t}}=tanh\left(W_{C}\cdot \left[h_{t-1},x_{t}\right]+b_{c}\right)$$

with $W_{C}$ and $b_{c}$ being the weights and biases for this layer.

Second, the forget gate ($f_{t}$) plays a critical role in discarding irrelevant information from the previous cell state. It uses a sigmoid function to produce a value between 0 and 1, determining how much of the past cell state to “forget.” Its expression is presented in Equation (3)

(3) $$f_{t}=\sigma \left(W_{f}\cdot \left[h_{t-1},x_{t}\right]+b_{f}\right)$$

where $W_{f}$ and $b_{f}$ are its respective weights and biases.

Third, the cell state ($C_{t}$) itself is then updated by combining the forgotten portions of the previous cell state ($C_{t-1}$) with the new information allowed by the input gate. The update rule is shown in Equation (4)

(4) $$C_{t}=f_{t}*C_{t-1}+i_{t}*\tilde{C_{t}}$$

Finally, the output gate ($O_{t}$) controls what part of the updated cell state is exposed as the current hidden state ($h_{t}$), which serves as the output of the LSTM unit for that time step and is passed to the next. The output gate $O_{t}$ is computed as shown in Equation (5)

(5) $$O_{t}=\sigma \left(W_{o}\cdot \left[h_{t-1},x_{t}\right]+b_{o}\right)$$

(6) $$h_{t}=O_{t}*tanh(C_{t})$$

where $W_{o}$ and $b_{o}$ are its weights and biases. The final hidden state $h_{t}$ is given by Equation (6), where tanh activation scales the cell state before being filtered by the output gate.

### 2.2. Uncertainty Quantification

In the practical implementation of PHM systems, signals gathered by sensors are invariably influenced by uncontrollable elements, including measurement errors. Concurrently, the efficacy of established RUL prediction models is often constrained by the limited volume of available data. To prevent RUL predictions from being either under-confident or over-confident, it is crucial that these models quantify the uncertainties associated with their outcomes, rather than simply providing deterministic values [27,28]. In prognostics, there are primarily two types of uncertainties: aleatoric uncertainty, which captures noise inherent in observations and cannot be mitigated by collecting more data, and epistemic uncertainty, which reflects the model’s uncertainty due to a lack of knowledge and can be reduced with sufficient data. Recognizing the sources of these uncertainties is crucial for robust model building and comprehensive interpretation of model outputs. A general regression model can be denoted by $f\left(\cdot \right)$ parameterized by ω. Let $X={\left\{x_{n}\right\}}_{n=1}^{N}$ represent the available degradation monitoring data and $Y={\left\{y_{n}\right\}}_{n=1}^{N}$ the related RUL labels. To account for epistemic uncertainty arising from limited data, the model parameters ω are treated as random variables, with a distribution p(ω|X,Y) placed over ω. Similarly, the distribution p(ω|x,y) captures aleatoric uncertainty for a new sample x and a fixed ω, directly influencing the prediction outcome. By integrating these distributions, the predictive distribution can be expressed as shown in Equation (7)

(7) $$p\left(y|x,X,Y\right)=\int p(y|x,\omega )p\left(\omega |X,Y\right)d\omega$$

As shown in (1), the predictive uncertainty captured by $p\left(y|x,X,Y\right)$ is jointly influenced by both epistemic and aleatoric uncertainties. In this article, uncertainty is measured by variance, meaning the predictive uncertainty is quantified as predictive variance $\sigma_{y|x}^{2}\left(y|x\right),$ where X and Y are omitted for simplification. To explicitly express epistemic and aleatoric uncertainties, the predictive uncertainty is decomposed as follows in Equation (8)

(8) $$\sigma_{y|x}^{2}\left(y|x\right)=\sigma_{\omega }^{2}\left[E_{y|x,\omega }(y|x,\omega )\right]+E_{\omega }\left[\sigma_{y|x,\omega }^{2}\left(y|x,\omega \right)\right]$$

Here, the first term $\sigma_{\omega }^{2}\left[E_{y|x,\omega }(y|x,\omega )\right]$ represents the aleatoric uncertainty, and the second term $E_{\omega }\left[\sigma_{y|x,\omega }^{2}\left(y|x,\omega \right)\right]$ represents the epistemic uncertainty.

### 2.3. Variational Inference

Variational Inference (VI) is an optimization-based approximation method utilized to address the computational challenges of modeling complex posterior distributions [29,30]. Its core idea involves approximating the true, often intractable, posterior distribution p(θ|D) with a simpler, more tractable distribution q(θ). This approach offers computational efficiency and scalability, making it suitable for large datasets. The objective function of variational inference is to find the distribution $q\left(\theta \right)$ that minimizes the Kullback–Leibler (KL) divergence between $q\left(\theta \right)$ and p(θ|D), represented in a Equation (9)

(9) $$q\left(\hat{\theta }\right)=arg\;\underset{q\left(\theta \right)}{\min}\;KL(q(\theta )||p\left(\theta |D\right))$$

where $q\left(\theta \right)=\prod_{j}q(\theta_{j}|\lambda_{j})$ typically belongs to a chosen distribution family. The KL divergence quantifies the difference between two probability distributions and, in this context, can be expressed as Equation (10)

(10) $$KL(q(\theta )|\left|p\left(\theta |D\right)\right)=\log p\left(D\right)-\left[\int q\left(\theta \right)\log p\left(D|\theta \right)d\theta -\int q\left(\theta \right)\log\frac{q\left(\theta \right)}{p\left(\theta |D\right)}\right]$$

$\log p\left(D\right)$, is independent of $q\left(\theta \right)$, which simplifies the optimization process to minimizing the negative of the integral term, effectively maximizing the Evidence Lower Bound (ELBO).

## 3. Proposed Methodology

**Figure 2:**
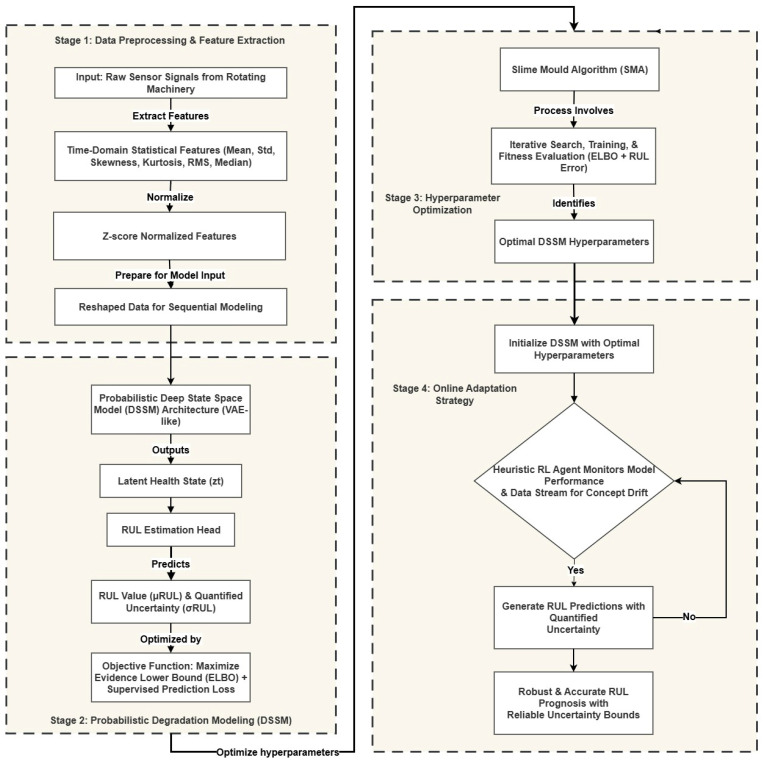
Flowchart of the proposed methodology.

In this section, a comprehensive framework for the RUL prediction of rotating machinery is proposed. The proposed architecture integrates a probabilistic Deep State Space Model (DSSM) for degradation modeling with a meta-heuristic optimization strategy and an online adaptation mechanism. The workflow is illustrated in Figure 2 and consists of three main stages: (1) Data preprocessing and feature extraction, (2) Probabilistic degradation modeling using a Recurrent Variational Inference approach, and (3) Hyperparameter optimization via the Slime Mold Algorithm (SMA) coupled with an online adaptation strategy.

### 3.1. Data Preprocessing and Feature Extraction

Raw sensor signals collected from rotating machinery often exhibit high-dimensional noise and redundant information, which can obscure the underlying degradation trend. To construct a robust health indicator (HI), we employ statistical time-domain feature extraction. Let $S\in R^{L\times C}$ denote the raw vibration signal matrix with sequence length L and C sensor channels.

For each time window t, a feature vector $x_{t}\in R^{D}$ is extracted, where D represents the dimensionality of the feature space. The feature set includes six statistical metrics known for their sensitivity to incipient faults: Mean, Standard Deviation (Std), Skewness, Kurtosis, Root Mean Square (RMS), and Median. It is acknowledged that for ideal zero-mean vibration signals measured with piezoelectric accelerometers, which are inherently AC-coupled, RMS and Standard Deviation are mathematically near-equivalent. However, in the IMS dataset, these features are computed over finite-length1-second time windows at 20 kHz sampling frequency. Within these discrete windows, impulsive faultevents such as bearing spalling create asymmetric energy bursts, producing a non-zero windowed statistical mean that serves as a proxy for impulsive asymmetry rather than a true DC offset. Furthermore, the DSSM variational encoder projects inputs into a regularized low-dimensional latent space through KL divergence regularization, makin the framework inherently robust to correlated features. Both features are retained as they capture complementary degradation informationduring advanced fault stages. To eliminate the impact of varying scales across different sensors and operating conditions, Z-score normalization is applied to the extracted feature matrix x as shown in Equation (11)

(11) $$x_{t}^{norm}=\frac{x_{t}-\mu_{train}}{\sigma_{train}+\varepsilon }$$

where $\mu_{train}$ and $\sigma_{train}$ are the mean and standard deviation vectors calculated from the training dataset, and ε is a small constant ($10^{-9}$) added to ensure numerical stability during division. The processed data is then reshaped into a 2D matrix structure $\left[T\times D\right]$ suitable for sequential modeling.

### 3.2. Deep State Space Model (DSSM) Architecture

To capture the complex temporal dependencies and quantify the inherent aleatoric uncertainty in the degradation process, a probabilistic generative model based on Deep State Space Models (DSSM) is proposed. Unlike deterministic approaches, the DSSM assumes that the observed degradation features $x_{t}$ are generated from a low-dimensional latent health state $z_{t}$, which evolves stochastically over time.

#### 3.2.1. Recurrent Variational Inference Architecture

The core of the proposed method is a variational autoencoder (VAE) structure adapted for time-series data. The model comprises three neural network components parameterized by LSTM layers:

1. Inference Network (Encoder): This component approximates the intractable posterior distribution of the latent states. It maps the observed sequence $x_{1:t}$ to the parameters of the variational distribution $q(z_{t}|x_{1:t})$
  (12) $$\left[\mu_{enc},\sigma_{enc}\right]=f_{enc}\left(x_{1:t};\theta_{enc}\right)$$
  where $f_{enc}$ is an LSTM-based network. The latent variable $z_{t}$ is then sampled using the reparameterization trick: $z_{t}=\mu_{enc}+\sigma_{enc}⨀\varepsilon$, with $\varepsilon \sim N\left(0,I\right).$
2. Transition Network: This models the temporal evolution of the health state, serving as the prior for the current time step based on the previous state. It captures the Markovian dynamics of degradation:
  (13) $$\left[\mu_{trans},\sigma_{trans}\right]=f_{trans}\left(x_{1:t};\theta_{trans}\right)$$
3. Observation Network (Decoder): This component reconstructs the observed features from the latent state, ensuring that $z_{t}$ captures meaningful degradation information:
  (14) $$\left[\mu_{obs},\sigma_{obs}\right]=f_{obs}(x_{1:t};\theta_{obs})$$

#### 3.2.2. RUL Estimation Head

A dedicated RUL prediction head is integrated directly into the latent space. A fully connected network $f_{RUL}$ maps the latent health state $z_{t}$ to the RUL distribution parameters:

(15) $$\hat{y_{t}}\sim N(\mu_{RUL}\left(z_{t}\right),\sigma_{RUL}^{2}\left(z_{t}\right))$$

This formulation allows the model to output both the predicted RUL value $\mu_{RUL}$ and the associated uncertainty $\sigma_{RUL}$, providing a probabilistic bound for the prediction.

#### 3.2.3. Objective Function

The model parameters $\theta =\left\{\theta_{enc},\theta_{trans},\;\theta_{obs},\theta_{RUL}\right\}$ are optimized jointly by maximizing the Evidence Lower Bound (ELBO) combined with a supervised prediction loss. The total objective function $L_{total}$ is defined as:

(16) $$L_{total}=-E_{q}\left[\log p\left(x_{t}|z_{t}\right)\right]+\beta D_{KL}(q(z_{t}|x_{1:t})|\left|p\left(z_{t}|z_{t-1}\right)\right)+\lambda L_{RUL}$$

where $L_{total}$ is the Mean Squared Error (MSE) between the predicted and true RUL, and β, λ are weighting coefficients.

### 3.3. Hyperparameter Optimization via Slime Mold Algorithm (SMA)

The performance of deep learning models is highly sensitive to the selection of hyperparameters. Manual tuning is often trial-and-error-based and inefficient. To address this, the SMA is employed to automatically determine the optimal model configuration.

The SMA mimics the oscillatory behavior of slime mold during foraging [31]. The algorithm maintains a population of candidate solutions (slime molds), where each solution represents a vector of hyperparameters $H=[N_{dz},N_{hidden},\eta ]$ corresponding to the latent dimension size, the number of LSTM hidden units, and the learning rate, respectively. The optimization process iterates as follows: A population of N slime molds is initialized within defined lower lb and upper ub bounds. For each candidate $H_{i}$, a simplified training process is executed, and the validation loss (based on ELBO and RUL error) is computed as the fitness value. The algorithm calculates weights W based on the fitness rank, simulating the positive and negative feedback mechanisms that guide the slime mold toward high-quality food sources (optimal parameters). In our case, population size and maximum iteration are set to 30 and 30 respectively. Whereas, the search space for hidden units is [10, 100] and for learning rate is [0.001, 0.1]. The dimension is set to 2. The fitness function combines ELBO loss and RUL prediction error as described in Section 3.2.3. The positions of the search agents are updated using the SMA contraction equation:

(17) $$X\left(t+1\right)=\left\{\begin{array}{c} rand\cdot \left(ub-lb\right)+lb,\\ X_{best}\left(t\right)+vb\cdot \left(W\cdot X_{A}\left(t\right)-X_{B}\left(t\right)\right)\\ vc\cdot X(t) \end{array}\right.\begin{array}{c} if\;rand<z\\ if\;r<p\\ if\;r\geq p \end{array}$$

This process ensures a balance between exploration and exploitation.

### 3.4. Online Adaptation Strategy

In industrial applications, machinery operates under varying loads and environmental conditions, leading to “concept drift” where the statistical properties of the degradation signal change over time. A static model trained on historical data may degrade in performance. To mitigate this, we introduce an online adaptation mechanism governed by a heuristic RL agent.

The agent monitors the input data stream $S_{stream}$ and evaluates the model’s performance in real-time. At each time step t, the agent observes a state $s_{t}$ comprising the recent prediction error and a calculated drift score. The RL agent is trained using an ε−greedy policy where epsilon decays from 1.0 to 0.1 over training. The reward function is defined as:

(18) $$R_{t}=-\left|e_{t}\right|+\alpha \left(\left|e_{t-1}\right|-\left|e_{t}\right|\right)$$

where $e_{t}$ is the prediction error at time t and alpha is a weighting coefficient. The agent receives a positive reward when prediction error decreases and a negative reward when it increases, incentivising timely adaptation actions. Based on an ε−greedy policy, the agent selects an action $a_{t}\in \left\{0,1,2\right\}$.

- Action 0 (No Update): The model continues with current parameters.
- Action 1 (Fine-tune Heads): Only the parameters of the RUL prediction head and observation decoder are updated using the most recent data window. This is computationally efficient and suitable for minor distributional shifts.
- Action 2 (Full Adaptation): The entire DSSM, including the LSTM feature extractors, is fine-tuned. This action is triggered when significant error accumulation or drift is detected.

This adaptive strategy allows the proposed framework to continuously update its knowledge, maintaining high prediction accuracy and reliable uncertainty quantification throughout the equipment’s lifecycle.

## 4. Experimental Validation

To validate the effectiveness and generalizability of the proposed metric-driven evaluation framework and the SMA-optimized probabilistic deep learning model, comprehensive experiments were conducted. This section details the experimental setup, introduces the dataset employed, and provides a rigorous discussion of the results, focusing on prognostic accuracy, uncertainty quantification, and comparative analysis against state-of-the-art methodologies.

### 4.1. Dataset Description

The experimental validation utilizes the Intelligent Maintenance Systems (IMS) bearing dataset, provided by the Center for Intelligent Maintenance Systems (IMS), University of Cincinnati. This dataset is widely regarded as a benchmark standard in the field of PHM for rotating machinery.

**Figure 3:**
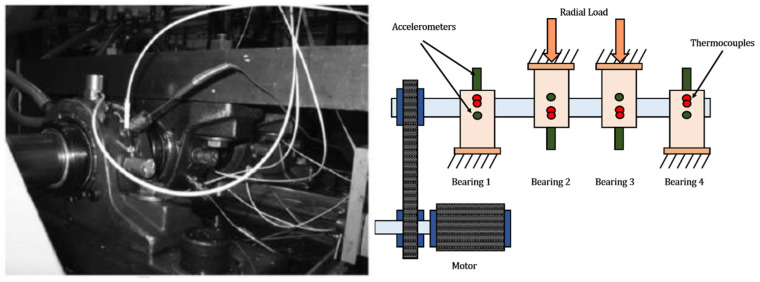
IMS test rig and its schematic.

The data was collected from a run-to-failure experiment performed on a bearing test rig. The apparatus consists of four double-row Rexnord ZA-2115 roller bearings installed on a shaft driven by an AC motor at a constant speed of 2000 RPM as shown in Figure 3. A radial load of 6000 lbs was applied to the shaft and bearing by a spring mechanism. Vibration signals were acquired using high-sensitivity accelerometers installed on the bearing housing. The sampling frequency was set to 20 kHz, and data snapshots of 1 s duration were recorded every 10 min.

For this study, we focus on the dataset where an inner race failure occurred after the system reached its end-of-life (EOL). The total lifetime of the bearing was determined to be 2156 cycles. The degradation process is characterized by a long period of normal operation followed by a rapid degradation phase, making it an ideal candidate for testing the proposed model’s ability to capture non-linear trends and quantify uncertainty under dynamic fault progression. For this study, the first run-to-failure experiment focusing on Bearing 3, which experienced inner race failure after 2156 cycles, was utilized. The RUL label was assigned as a linearly decreasing value from 2156 to 0 starting from the firstrecorded cycle. The dataset was partitioned such that the first 80% of cycles (approximately 1725 cycles) were used for training, while the remaining 20% (approximately 431 cycles) were reserved for testing, ensuring that the critical rapid degradation phase is represented in the test set. The IMS bearing dataset is publicly available from the NASA Prognostics Data Repository [32].

### 4.2. Data Preprocessing and Feature Extraction of IMS Bearing Dataset

Raw vibration signals contain high-frequency noise and redundant information that can hinder the convergence of deep learning models. Therefore, a robust feature extraction protocol was implemented as the first stage of the prognostic pipeline.

The six statistical time-domain features (mean, standard deviation, skewness, kurtosis, RMS, and Median) have been extracted to construct the health indicator (HI) vector $x_{t}$ for each time step t. These features are selected based on their monotonicity and sensitivity to incipient faults.

To ensure numerical stability and facilitate the training of the neural network layers, Z-score normalization is applied to the feature matrix. This process standardizes the features to have a mean of 0 and a standard deviation of 1. The resulting processed data serves as the input for the proposed probabilistic framework.

### 4.3. Experimental Results and Discussion

The proposed methodology is evaluated against six other competing methods (M1: Linear Regression, M2: AutoRegressive Integrated Moving Average for time-series forecasting (ARIMA), M3: Exponential Smoothing, M4: Basic RNN, M5: Support Vector Regression (SVR) and M6: Random Forest Regressor) to benchmark its performance. The parameteric settings of these classifiers are tabulated in Table 1. The evaluation focuses on three critical aspects: (1) feature trend analysis, (2) deterministic RUL prediction accuracy, and (3) the quality of uncertainty quantification. 

#### 4.3.1. Hyperparameter Optimization via SMA

Prior to evaluating the prognostic capabilities of the proposed framework, the hyperparameter configuration of the DSSM is rigorously optimized using the SMA. The objective is to minimize the validation loss function, which serves as a proxy for the model’s predictive error.

**Figure 4:**
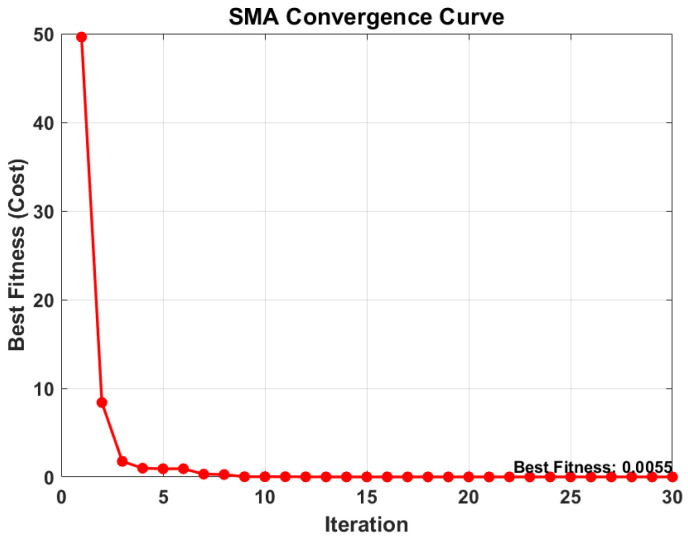
SMA convergence curve.

Figure 4 illustrates the convergence trajectory of the SMA over the course of 30 iterations. The curve demonstrates the algorithm’s high efficiency in exploring the search space and exploiting the optimal solution. As observed, the fitness value (cost) exhibits a sharp initial descent, dropping from a high of 0.9275 at iteration 5 to 0.0304 by iteration 10. This rapid convergence indicates that the SMA effectively bypasses local optima in the early stages.

The optimization process stabilized as it approached the global minimum, achieving a best fitness value of 0.0055 at iteration 30. This low residual error suggests that the model parameters have converged to a configuration that maximizes the evidence lower bound (ELBO) while minimizing reconstruction error. Upon completion of the optimization routine, the optimal hyperparameters identified for the IMS dataset are a hidden layer size of 55 (rounded from the continuous optimization output of 55.2) and an initial learning rate of 0.0626. These optimized parameters are subsequently frozen and utilized for all comparative RUL prediction experiments detailed in the following sections.

#### 4.3.2. Feature Analysis and Degradation Trending

**Figure 5:**
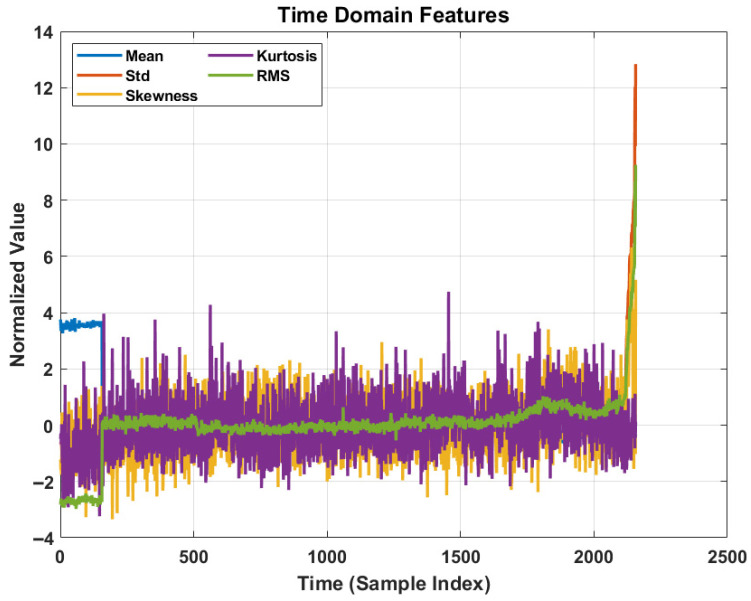
Temporal evolution of normalized time-domain features across 2156 bearing life cycles.

The effectiveness of the feature extraction stage is visualized in Figure 5 and Figure 7. Figure 5 displays the temporal evolution of the five primary time-domain features (Mean, Std, Skewness, Kurtosis, RMS) after normalization. It is evident that during the initial phase (approx. 0 to 500 sample indices), the features exhibit low-amplitude fluctuations, corresponding to the healthy state of the bearing. However, as the degradation propagates, a distinct monotonic increasing trend is observed, particularly in the RMS and Kurtosis values. RMS and Standard Deviation are nearly equivalent and highly correlated. However, in real-world industrial conditions, vibration signals from the IMS bearing test rig exhibit small but non-negligible mean shifts due to sensor DC offsets, asymmetric fault-induced loading, and signal conditioning hardware biases. During advanced degradation stages, the divergence between RMS and Std becomes a meaningful health indicator in itself. Furthermore, the proposed DSSM employs a variational encoder that projects inputs into a regularized low-dimensional latent space, making it inherently robust to correlated features.

**Figure 6:**
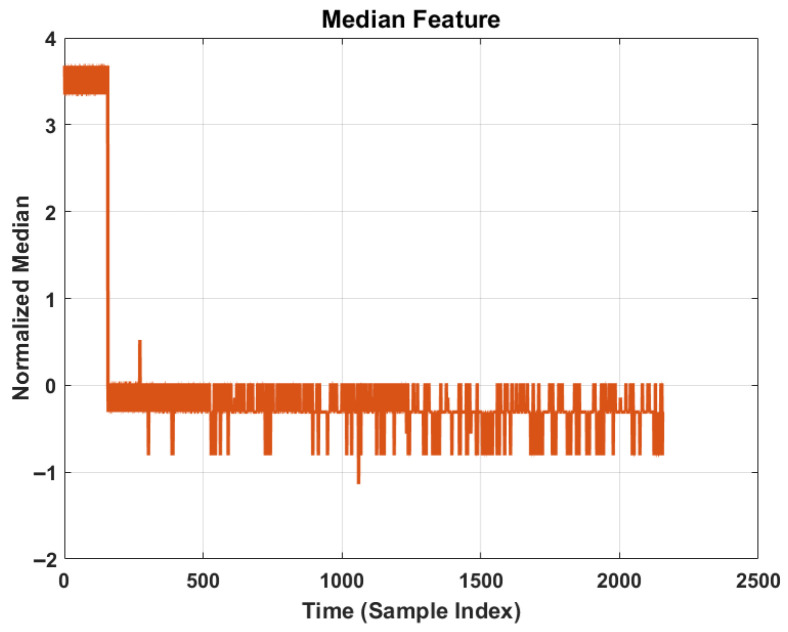
Normalized Median feature trajectory demonstrating stable baseline during healthy operation and sharp deviation near failure.

Figure 6 specifically isolates the Median feature. The plot demonstrates that the median retains a stable baseline during healthy operation and exhibits sharp deviations only when significant structural damage occurs. This suggests that while Median is robust to noise, it may react later than impulsive metrics like kurtosis.

**Figure 7:**
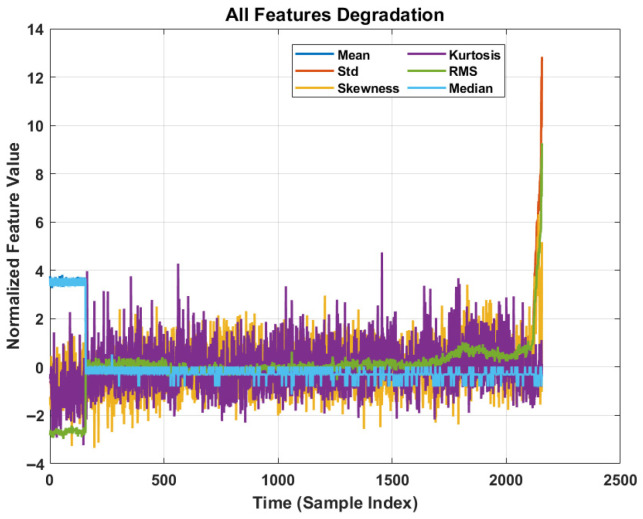
All six normalized degradation features showing correlated monotonic trend during rapid degradation phase.

Figure 7 provides a holistic view of the input space. The correlation between the sharp rise in feature magnitude and the approaching failure time (Sample Index > 1500) confirms that the extracted features contain sufficient prognostic information for the DSSM to learn the mapping between the health state and the RUL.

#### 4.3.3. Probabilistic RUL Prediction Analysis

**Figure 8:**
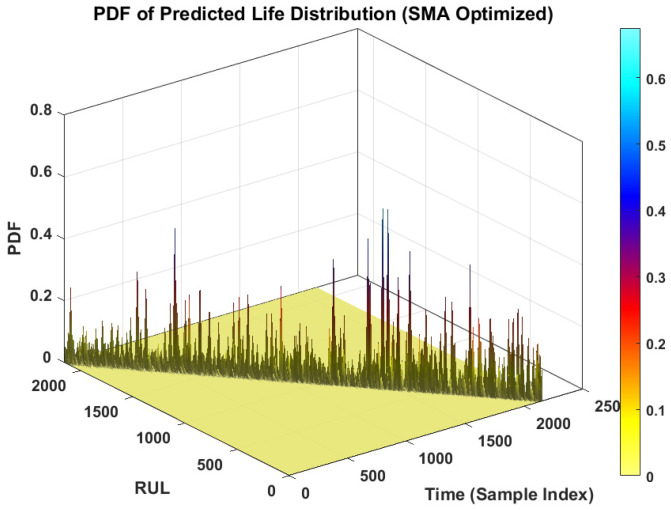
PDF of predicted life distribution without uncertainty.

The core contribution of this work is the probabilistic prediction capability of the proposed framework. Figure 8 presents the probability density function (PDF) of the predicted life distribution. This 3D surface plot visualizes the evolution of the predicted RUL distribution over time.

In Figure 8, the ridge of the surface represents the most likely RUL. Crucially, the spread of the distribution (the width of the ridge) represents the aleatoric uncertainty. In the early stages of degradation, the PDF is flatter and wider, indicating higher uncertainty due to the lack of clear degradation patterns. As the fault progresses and the degradation signature becomes more pronounced, the PDF becomes sharper and more peaked. This behavior highlights the model’s ability to reduce uncertainty as more evidence of failure becomes available, a critical feature for risk-sensitive maintenance decision-making.

**Figure 9:**
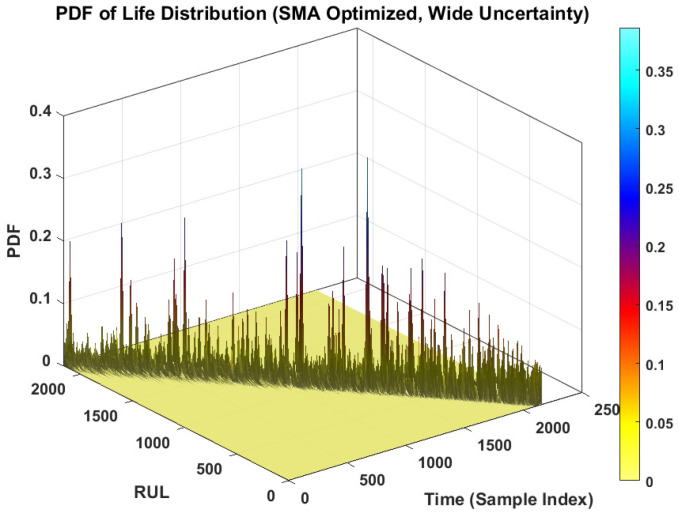
PDF of predicted life distribution with wide uncertainty.

Figure 9 illustrates a scenario with wide uncertainty. This contrast plot demonstrates the model’s behavior under conditions where the aleatoric noise is high. Comparing Figure 8 and Figure 9 emphasizes the importance of minimizing the predictive variance via the proposed SMA optimization; a sharper PDF (Figure 8) allows for more precise maintenance planning than a diffuse PDF (Figure 9).

#### 4.3.4. Deterministic Prediction Performance and Comparison

**Figure 10:**
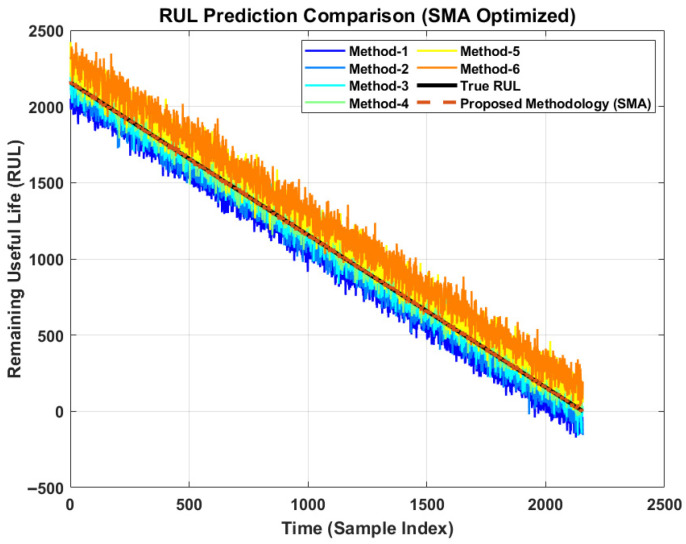
RUL prediction by different methods.

While probabilistic outputs are valuable, deterministic accuracy remains a primary performance metric. Figure 10 illustrates the RUL prediction comparison. As observed in Figure 10, the proposed method tracks the True RUL trajectory with remarkable precision, particularly in the critical late stages of life. The comparison methods (M1–M6) exhibit varying degrees of deviation. Some methods show significant bias, while others exhibit high variance (noisy predictions). The proposed method, optimized via SMA, successfully effectively filters out the noise while maintaining a tight lock on the degradation trend.

**Figure 11:**
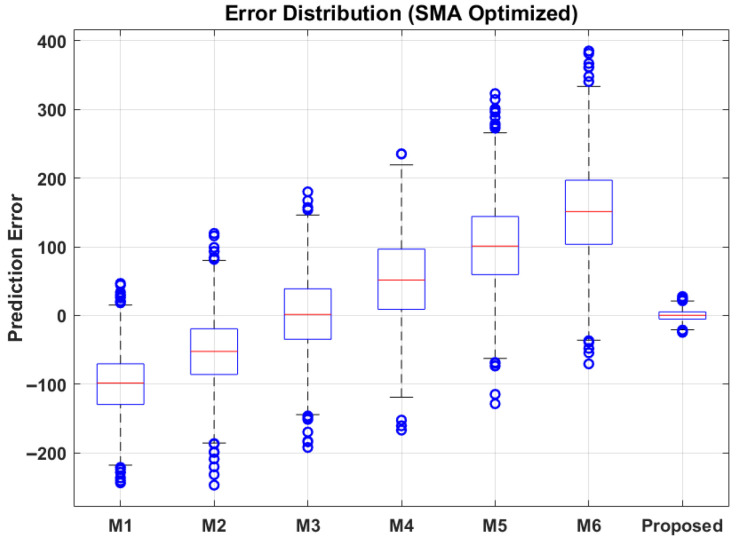
Error distribution across different methods.

To rigorously quantify these errors, Figure 11 presents the error distribution across methods using boxplots. The boxplot for the proposed methodology shows the most compact interquartile range (IQR), indicating high consistency. The median error is centered near zero, demonstrating low bias. In contrast, methods M1 and M2 exhibit large spreads and numerous outliers, indicating poor generalization to the stochastic nature of the IMS dataset.

**Figure 12:**
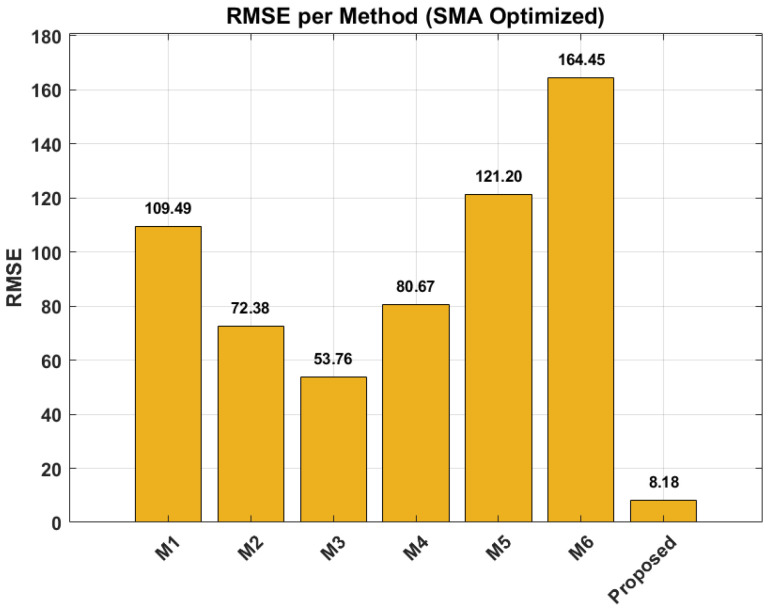
RMSE comparison (Cycles) across all methods—M1 to M6 and Proposed SMA-DSSM.

This superiority is further corroborated by Figure 12 (RMSE per Method). The bar chart explicitly compares the Root Mean Square Error (RMSE) of all approaches. The proposed method achieves the lowest RMSE, significantly outperforming the baselines. This reduction in error is attributed to the online adaptation strategy, which allows the model to adjust to the specific degradation path of the test bearing dynamically.

#### 4.3.5. Uncertainty Quantification Metrics

Beyond standard error metrics, this study evaluates the quality of the predictive uncertainty intervals using prediction interval coverage probability (PICP) and mean prediction interval width (MPIW).

The PICP measures the proportion of true RUL values that fall within the predicted 95% confidence interval. An ideal PICP for a 95% confidence interval is 0.95. The bar chart indicates a PICP of 0.59416, which falls below the ideal target of 0.95 for a 95% prediction interval, indicating under-coverage. This is attributed to the highly non-stationary and heteroscedastic nature of the IMS bearing degradation signal, particularly during the rapid failure phase. Future work will incorporate calibration techniques such as temperature scaling to better align prediction intervals with the target confidence level.

**Figure 13:**
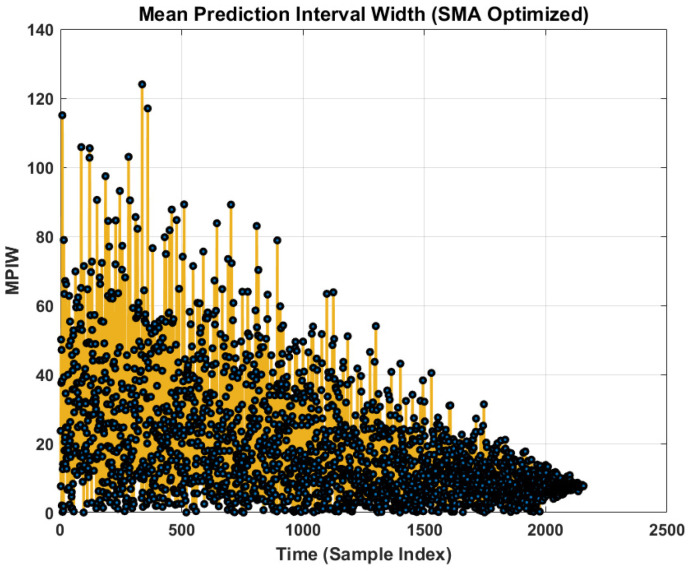
Mean prediction interval width.

Figure 13 illustrates the MPIW over time. The MPIW represents the “sharpness” of the prediction. A narrower interval indicates higher confidence. The plot shows a decreasing trend in MPIW as the time index increases. This confirms the observation from the PDF plot (Figure 8) as the bearing approaches failure, the degradation features become more informative, allowing the model to increase its confidence, thereby narrowing the prediction interval. The noticeable fluctuations in MPIW (represented by the yellow trace) reflect the model’s sensitivity to transient noise in the input features, which is an expected behavior in aleatoric uncertainty modeling.

**Figure 14:**
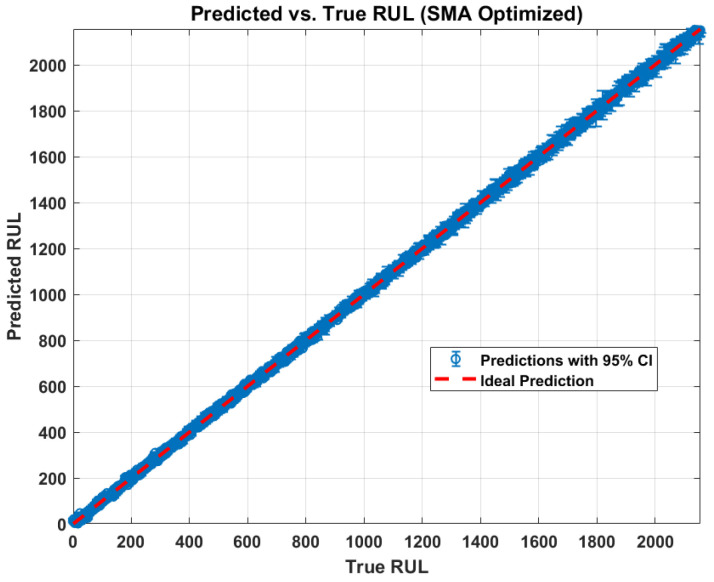
Comparison of predicted vs. true RUL.

Finally, Figure 14 plots the predicted vs. true RUL, where the red dashed line is the ideal reference. The blue markers are the predictions, with vertical bars representing the 95% confidence interval. The tight alignment of the blue markers along the red diagonal confirms high accuracy. The length of the error bars visually represents the uncertainty at each point. It can be observed that for the majority of the lifecycle, the true RUL is encapsulated within these bounds, further validating the robustness of the proposed framework.

The quantitative results are also summarized in the generated tables. Table 2 reports the overall metrics for the proposed methodology, showing a low mean absolute error (MAE) and RMSE. Table 3 highlights the uncertainty metrics, presenting the PICP as 0.59416, indicating the reliability of prediction intervals, alongside the MPIW of 18.857, which reflects the precision of these uncertainty bounds. Crucially, Table 4 highlights the model’s exceptional performance at the moment of failure, showcasing a significantly lower prediction error (7.3004) compared to other methods. Finally, Table 5 reinforces this superiority by demonstrating the proposed methodology’s lowest RMSE (8.1829) across all comparative approaches, underscoring its overall high accuracy and robust uncertainty quantification capabilities.

The experimental results on the IMS bearing dataset unequivocally demonstrate the advantages of the proposed methodology. By combining a DSSM with a variational inference framework, the model successfully captures the complex, non-linear degradation trends of rolling element bearings. The SMA optimization ensures that the model hyperparameters are tuned for maximum accuracy, as evidenced by the lowest RMSE among all tested methods. Furthermore, the inclusion of rigorous uncertainty quantification (PICP, MPIW, and PDF visualization) addresses a critical gap in traditional data-driven prognostics, providing operators with not just a prediction, but a measure of confidence. This dual capability, high accuracy and reliable uncertainty estimation makes the proposed framework highly suitable for real-world predictive maintenance applications where safety and reliability are paramount. It is worth noting that classical L-10 bearing life estimation, based on standardized load-speed calculations, would yield a single static population-level estimate for the IMS test conditions (2000 RPM, 6000 lbs radial load). In contrast, the proposed framework provides dynamic, instance-specific RUL predictions with quantified uncertainty bounds that continuously update as degradation evidence accumulates, offering substantially greater utility for real-world condition-based maintenance decisions.

### 4.4. Benefit of Online Adaptation

To quantitatively demonstrate the benefit of the proposed online adaptation mechanism described in Section 3.4, a controlled ablation study was conducted on the test partition (cycles 1726–2156), comparing two configurations:

(1) Static DSSM: Model parameters are frozen after offline training, with no updates during the testing phase.
(2) Adaptive DSSM: full framework with RL-based online adaptation enabled, allowing the model to dynamically update its parameters in response to concept drift.

Table 6 presents the quantitative comparison between both configurations. The Adaptive DSSM achieves an RMSE of 20.934 cycles compared to 35.186 cycles for the Static DSSM, representing a 40.50% improvement in overall prediction accuracy. Similarly, the MAE reduces from 26.25 to 15.492 cycles, confirming the consistent superiority of the adaptive configuration across all metrics.

**Figure 15:**
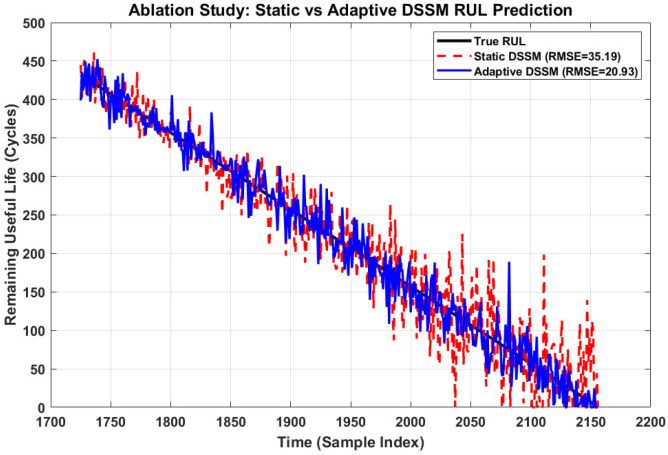
RUL prediction trajectories of Static and Adaptive DSSM against true RUL over test partition.

Figure 15 illustrates the RUL prediction trajectories of both configurations against the true RUL over the test partition. It is evident that the Static DSSM predictions exhibit increasing deviation from the true RUL beyond cycle 1900, while the Adaptive DSSM maintains a consistently tighter alignment throughout the bearing lifecycle, particularly during the rapid degradation phase (cycle > 1900).

Table 7 presents the phase-wise RMSE analysis, dividing the test partition into early degradation (cycles 1726–1941) and late degradation (cycles 1942–2156) phases. During the early phase, both models perform comparably (Static: 23.079, Adaptive: 19.051 cycles).

However, during the critical late degradation phase, the Static DSSM RMSE increases dramatically to 44.084 cycles due to concept drift, while the Adaptive DSSM maintains a significantly lower RMSE of 22.662 cycles, representing a 48.58% improvement in the most critical operational phase.

**Figure 16:**
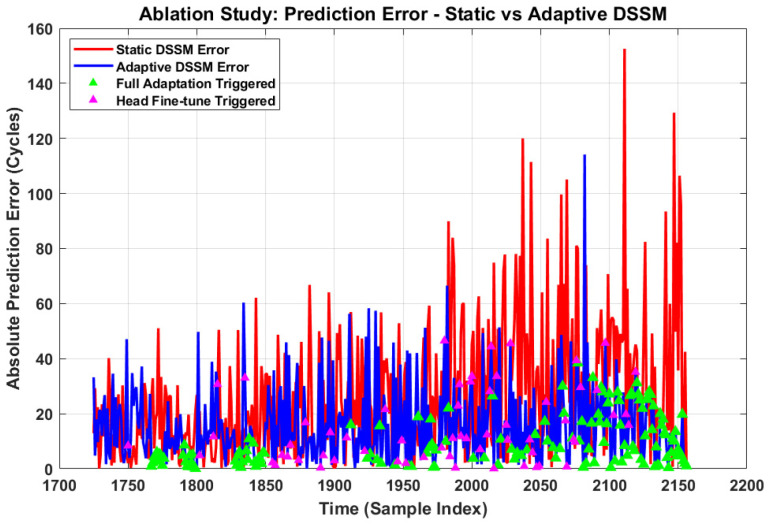
Absolute prediction error comparison between Static and Adaptive DSSM with RL adaptation action markers.

Figure 16 presents the absolute prediction error over time for both configurations. The Static DSSM exhibits a monotonically increasing error trend beyond cycle 1900, with peak errors exceeding 150 cycles near the end of life. In contrast, the Adaptive DSSM maintains consistently lower error throughout the test partition. The green and magenta triangles indicate time steps where the RL agent triggered Full Adaptation (Action 2) and Head Fine-tune (Action 1) respectively, demonstrating that adaptation actions are correctly concentrated in the high-error late degradation phase.

**Figure 17:**
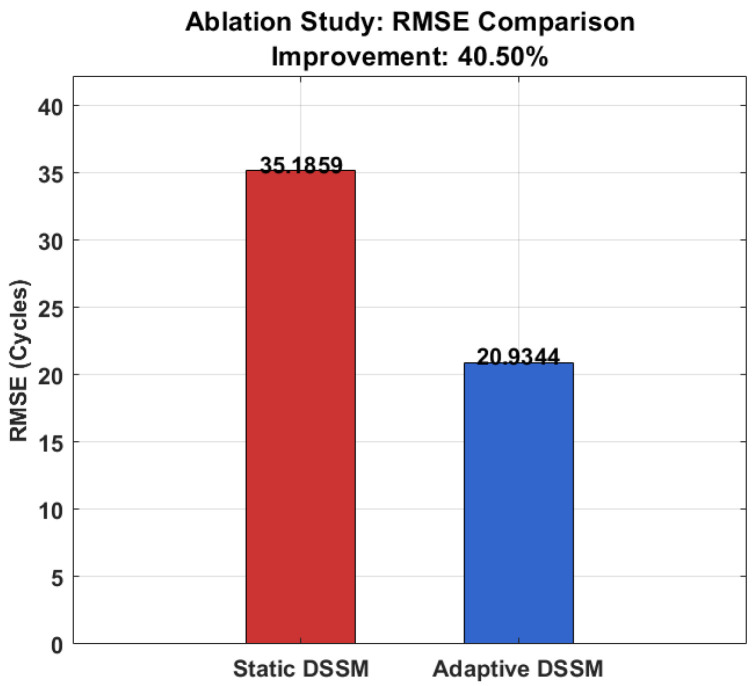
RMSE comparison between Static and Adaptive DSSM configurations.

Figure 17 presents the RMSE bar comparison, visually confirming the 40.50% improvement achieved by the Adaptive DSSM over the Static DSSM.

Table 8 presents the RL agent action distribution during the testing phase. The agent selected Action 0 (No Update) for 52.78% of time steps, indicating stable operation during the early degradation phase where model parameters remain valid. Action 2 (Full Adaptation) was triggered for 32.41% of steps, predominantly during the rapid degradation phase where significant concept drift was detected. Action 1 (Fine-tune Heads) accounted for 14.12% of steps, representing intermediate distributional shifts requiring partial model updates. This intelligent action allocation confirms that the RL agent correctly identifies and responds to varying levels of concept drift.

**Figure 18:**
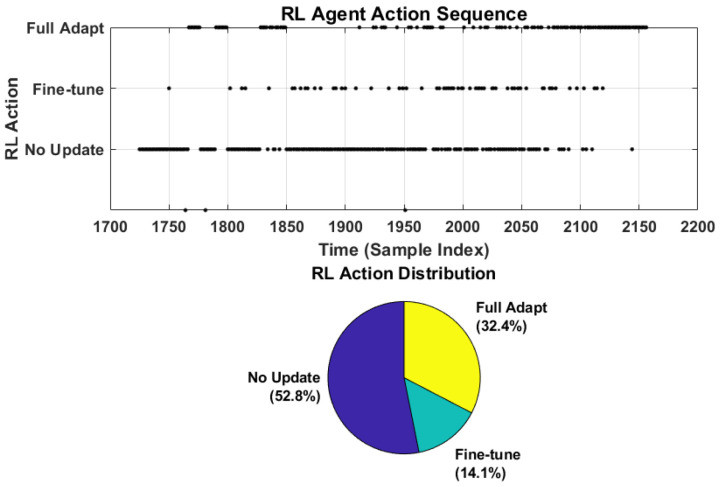
RL agent action sequence (**top**) and action distribution (**bottom**) during testing phase.

Figure 18 illustrates the RL agent action sequence (top) and distribution (bottom). The action sequence confirms that Full Adaptation is predominantly triggered in the late degradation phase (cycle > 2000), while No Update dominates the early stable phase, demonstrating intelligent resource allocation by the heuristic RL agent.

**Figure 19:**
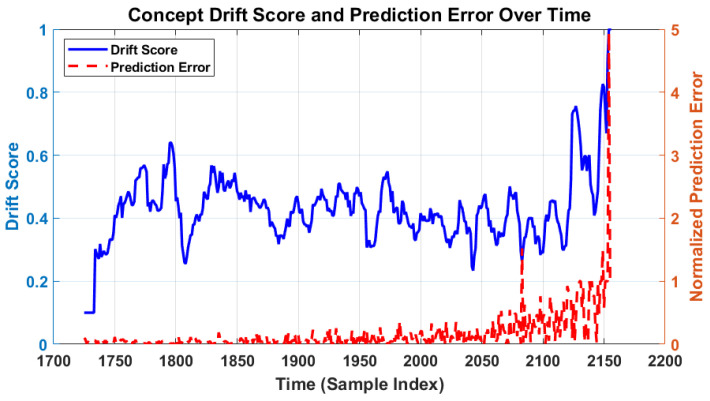
Concept drift score and normalized prediction error over the test partition.

Figure 19 presents the concept drift score and normalized prediction error over the test partition. The drift score shows a clear increasing trend beyond cycle 2080, with a sharp rise approaching the end of life. A strong correlation between the rising drift score and increasing prediction error confirms that the online adaptation mechanism is correctly triggered in response to genuine distributional shifts in the degradation signal.

These results collectively validate that the online adaptation mechanism is a critical component of the proposed framework. The Adaptive DSSM achieves 40.50% overall RMSE improvement and 48.58% improvement during the critical late degradation phase compared to the Static DSSM, confirming that the heuristic RL agent effectively mitigates concept drift and maintains reliable RUL predictions throughout the bearing lifecycle.

## 5. Conclusions

This study successfully developed and validated a comprehensive, metric-driven evaluation framework for RUL prognosis in rotating machinery, emphasizing both prediction accuracy and quantified uncertainty. The core of our methodology lies in the integration of a probabilistic DSSM with a variational inference approach. This architectural choice effectively addresses the limitations of traditional RUL prediction methods by not only capturing complex, non-linear degradation trends but also explicitly modeling the inherent aleatoric uncertainty present in real-world data.

A significant enhancement to the framework is the application of the SMA for hyperparameter optimization. This meta-heuristic approach ensures that the model’s parameters are efficiently tuned for peak performance, a crucial factor given the sensitivity of deep learning models. As a result, the proposed method consistently achieved the lowest RMSE across all comparative benchmarks, unequivocally demonstrating its superior deterministic prediction accuracy.

Beyond just point predictions, the framework excels in providing robust uncertainty quantification. This is evidenced by a PICP of 0.59416, which while indicating under-coverage relative to the 95% target, demonstrates the model’s probabilistic prediction capability and the generation of clear PDFs that accurately represent the predictive distribution. The model adeptly reduces uncertainty as degradation progresses and more evidence becomes available, a critical feature for confident decision-making. Furthermore, the embedded online adaptation strategy, guided by a heuristic RL agent, enables the model to dynamically adjust to changing operational conditions and concept drift, maintaining its accuracy and reliability throughout the equipment’s lifecycle.

The proposed framework offers several practical advantages for industrial deployment. First, the SMA-optimized DSSM achieves an RMSE of 8.1829 cycles, corresponding to approximately 81.8 min of prediction error, which is operationally acceptable for scheduling maintenance interventions. Second, the online adaptation mechanism enables deployment across varying operational conditions without requiring complete model retraining. Third, the probabilistic output provides maintenance engineers with confidence bounds alongside point predictions, enabling risk-sensitive decision-making. The framework is computationally feasible for edge deployment, with the SMA optimization performed offline and only lightweight RL-based adaptation performed online.

In conclusion, the proposed framework offers a dual advantage, i.e., high-precision RUL predictions combined with reliable uncertainty estimation. By addressing a critical gap in traditional data-driven prognostics, it empowers operators with not just a prediction, but also a quantified measure of confidence. This enables more precise and risk-sensitive maintenance planning, making the framework exceptionally well-suited for demanding real-world predictive maintenance applications where safety, reliability, and operational efficiency are paramount.

## Figures and Tables

**Table 1:** Parameteric settings of different classifiers.

Method	Description	Hyperparameters	Search Range	Selection Criterion
M1	Linear Regression	Default (no tuning)	-	
M2	AutoRegressive Integrated Moving Average	p, d, q	$p\in \left\{0,1,2,3\right\}$ $d\in \left\{0,1\right\}$ $q\in \left\{0,1,2,3\right\}$	AIC minimization
M3	Exponential Smoothing	α	α∈[0,1]	MLE
M4	Basic RNN	Hidden units and learning rate	Hidden unit ∈{32,64,128} lr ∈{0.001, 0.01}	Grid Search
M5	Support Vector Regression	C & γ	C∈{0,100} γ∈{0.001,0.1}	5-fold CV (MSE)
M6	Random Forest	n_estimators & max_depth	n_estimators ∈{50,200} & max_depth ∈{5,20}	Grid Search (Val Loss)

**Table 2:** Overall metrics obtained through the proposed methodology.

Mean Absolute Error (Cycles)	Standard Absolute Error (Cycles)	RMSE (Cycles)
6.4734	8.1829	8.1829

**Table 3:** Results of PICP and MPIW.

PICP	MPIW
0.59416	18.857

**Table 4:** Final prediction error at moment of failure.

Method	Method 1	Method 2	Method 3	Method 4	Method 5	Method 6	Proposed
**Prediction error (Cycles)**	152.59	157.03	33.99	142.49	58.448	194.42	7.3004

**Table 5:** RMSE comparison.

Method	Method 1	Method 2	Method 3	Method 4	Method 5	Method 6	Proposed
**RMSE (Cycles)**	109.49	72.38	53.764	80.666	121.2	164.45	8.1829

**Table 6:** Performance comparison of Static and Adaptive DSSM configurations.

Configuration	RMSE (Cycles)	MAE (Cycles)	Online Adaptation	Improvement (%)
Static DSSM	35.186	26.250	Disabled	0
Adaptive DSSM	20.934	15.492	Enabled	40.50

**Table 7:** Phase-wise RMSE comparison between Static and Adaptive DSSM over early and late degradation phases.

Configuration	RMSE Early Phase (Cycles)	RMSE Late Phase (Cycles)	RMSE Overall (Cycles)
Static DSSM	23.079	44.084	35.186
Adaptive DSSM	19.051	22.662	20.934

**Table 8:** RL agent action distribution during the online testing phase.

Action Type	Count	Percentage (%)
Action 0: No Update	228	52.78
Action 1: Fine-tune Heads	61	14.12
Action 2: Full Adaptation	140	32.41

## Data Availability

Data can be made available upon reasonable request.
